# Aging Processes of Working Memory in Different Modalities

**DOI:** 10.3390/neurolint16050084

**Published:** 2024-09-30

**Authors:** Ohad Levi, Eyal Heled

**Affiliations:** 1 Department of Psychology, Faculty of Social Sciences and Humanities, Ariel University, Ariel 4077625, Israel; levi1ohad1i@gmail.com; 2 The Rehabilitation Hospital, Sheba Medical Center, Ramat Gan 5262160, Israel

**Keywords:** working memory, elderly, tactual span, digit span, visuospatial span, aging

## Abstract

**Background**: Working memory (WM) involves temporarily storing and manipulating information. Research on the impact of aging on WM has shown inconsistent results regarding the decline in visual and verbal WM, with a lack of studies on tactile WM. This study aimed to assess the effects of aging on WM across verbal, visuospatial, and tactile modalities using span tasks of forward (storage) and backward (manipulation) stages. **Methods**: A total of 130 participants, divided into four age groups of 20–29, 60–69, 70–79, and 80–89, completed the Digit, Visuospatial, and Tactual Spans. Performance was analyzed using a 3 (Task) × 4 (Group) × 2 (Stage) mixed design repeated measures ANOVA. **Results**: The analysis revealed significant main effects for modality (*p* < 0.001, η_p_^2^ = 0.15), age (*p* < 0.001, η_p_^2^ = 0.48), and stage (*p* < 0.001, η_p_^2^ = 0.30). Digit Span outperformed the other modalities, while Tactual Span showed the worst performance. Additionally, task performance declined with age, and the forward stage was superior to the backward stage. Interaction effects indicated that Digit Span was less affected by aging compared to the Visuospatial and Tactual Spans (*p* = 0.004, η_p_^2^ = 0.07). Post hoc analyses further revealed that the Digit Span consistently outperformed the other modalities in both stages, with more pronounced differences observed in the forward stage. **Conclusions**: Verbal WM is more resilient to aging compared to the other modalities while tactile WM declines with age in a manner similar to verbal and visuospatial WM, suggesting a modality-specific impact of aging on WM.

## 1. Introduction

Working memory (WM) is defined as a complex neurocognitive capacity that allows simultaneous storage and manipulation of a limited amount of information in a short time [1,2,3]. WM is deemed crucial for many cognitive abilities in daily life, including learning, attention, mathematical thinking, and language comprehension [4,5].

Research on WM development across the lifespan has demonstrated a trend toward improvement until young adulthood, indicating that WM reaches its peak capacity in the age range of 20–35 years. Then, in middle adulthood (35–54 years), a moderate decline begins, which becomes more pronounced in late adulthood [6,7,8,9,10]. Nevertheless, some studies show that such a modest decay starts no sooner than in old age [11,12]. Furthermore, within the investigation of the aging effect on WM, another aspect explored is the performance of WM in different modalities, which has shown inconsistent results. While some studies have found that WM in the verbal modality appears to be relatively stable with aging, visuospatial WM exhibits a more pronounced gradual decline [10,11,12,13,14]. However, Vecchi et al. [15] revealed the opposite, showing a decrease limited only to verbal but not to visuospatial WM, and yet others point to a uniform aging trend of deterioration in verbal and visuospatial modalities [16,17,18]. These discrepancies suggest a lack of consensus regarding the trajectory of WM decline across modalities in old age, although it seems that there is a trend toward visuospatial deterioration, compared to verbal WM.

Fournet et al. [19] explained the superiority of verbal WM by suggesting that visuospatial WM relies more heavily on executive processes than verbal WM, rendering it more vulnerable to the effects of aging. Moreover, brain imaging studies have concluded that visuospatial WM deterioration is due to neurological depletion in the right hemisphere, compared to the left hemisphere, which is responsible for verbal functions [20,21,22,23]. Further studies have supported this claim, showing that primary visual processing areas (e.g., the prefrontal cortex) exhibit under-activation in late adulthood compared to earlier ages [22,24].

Interestingly, another explanation suggested to account for the aging effect on WM in different modalities was related to classical music exposure. This research showed a deterioration in verbal WM, whereas in visuospatial WM, an inconsistent positive impact was found, depending on the type of music presented [25,26]. Such findings demonstrate that exposure to external stimulation such as music can influence executive functions, possibly due to the neural systems shared between music and visuospatial processing [27].

In exploring storage versus manipulation components of WM aging in verbal and visuospatial modalities, Wingfield et al. [28] found a more pronounced age gap in verbal WM manipulation abilities than in storage between older adults and those aged 17–21, with the former exhibiting significantly poorer manipulation capacity. They further divided the older adults into young seniors (55–70 years) and older seniors (70+ years), revealing no significant subgroup differences in verbal storage, but significantly poorer manipulation performance in the older seniors group. They concluded that as opposed to storage, manipulation of verbal but not visual WM declines with age.

While much attention has been given to the verbal and visuospatial modalities, tactile WM has been investigated to a far lesser extent in the elderly population, even though it constitutes a significant part of WM functioning [29,30,31,32,33,34,35,36] and is associated with various cognitive abilities, such as memory, perception, and attention [30,31,35,36,37].

Tactile WM refers to the ability to maintain and manipulate tactile information temporarily [38]. Our literature review revealed no studies on tactile WM in the elderly population, except for a recent one by Heled and Levi [34], who evaluated WM function across verbal, visuospatial, and tactile modalities in four age groups of 8–9, 11–12, 25–35, and 60–69. They found that older adults showed poorer performance on the tactile compared to visuospatial and verbal WM tasks and argued for a modality-specific aging effect on WM.

In light of the inconsistent findings regarding age-related changes in WM across modalities [11,14] and the lack of research on tactile WM in old age [34], the first aim of our study was to examine the effect of aging on WM functioning across the verbal, visuospatial, and tactile modalities. This approach aimed to uncover age-related nuances in WM across these three modalities, potentially identifying which of them is most susceptible to aging effects.

Additionally, comparing storage and manipulation as separate components of WM indicates their distinct cognitive processes in WM function, as backward recall is more active and relies on executive processes [39,40]. Therefore, our second objective was to test how aging impacts the storage and manipulation components.

## 2. Materials and Methods

### 2.1. Participants

The requisite sample size was determined using G*Power analysis software (Version 3.1.9.7; [41]), initially indicating a minimum of 76 participants. However, to enhance statistical power, a larger sample size was employed. This study involved 130 participants (82 females), including 90 older adults (71 females) aged 60–89 years divided into three groups, with an additional group of 30 young adults (11 females) aged 20–29. Elderly participants were recruited from ads on social networks (e.g., Facebook, WhatsApp) and from nursing homes by introducing the study in their weekly meetings and scheduling those who agreed to participate. Young adult participants were recruited through social media and word of mouth or as part of course requirements for completing their bachelor’s degree. Non-student participants received a modest gift. Exclusion criteria encompassed a history of psychiatric or neurological disorders (e.g., epilepsy, stroke), sensory or motor impairments precluding computer use, and a diagnosis of a learning disability or developmental disorder. Inclusion criteria mandated right-hand dominance, a minimum of 12 years of education, and a Montreal Cognitive Assessment score of 26 or higher for the elderly group. The study protocol was approved by the Ethics Committee of Ariel University (AU-EH-20181220).

### 2.2. Instruments and Measures

#### 2.2.1. Demographics and Clinical Questionnaire

The demographic data were collected from participants utilizing a self-report questionnaire developed for the study, providing information on age, sex, years of education, native language, and handedness.

#### 2.2.2. Digit Span

The computerized Digit Span task was adapted from the Wechsler Adult Intelligence Test—3rd edition (WAIS-III; [42]). For forward recall, verbal stimuli consisting of sequences of numbers were presented at a rate of one stimulus per second. Participants were instructed to reproduce the sequences verbally in the same order as presented. The task commenced with two-digit sequences, incrementing by one digit upon correct response until the participant failed to recall two trials of the same length accurately. In the backward recall, the stimuli presentation was identical; however, participants were required to reproduce the sequences in reverse order. The dependent variable was the longest sequence the participants recalled in each stage separately.

#### 2.2.3. Tactual Span

For the Tactual Span task [32], which assesses tactile WM, participants positioned four fingers of each hand on a row of computer keyboard keys while blindfolded. In the forward recall, the examiner touched the participant’s knuckles in a predetermined sequence for one second each. Then, participants were instructed to reproduce the sequence by pressing the corresponding keys on the keyboard. Sequence lengths, consisting of three trials each, commenced with two stimuli and were incremented by one stimulus upon accurate recall in at least one trial, up to a maximum of nine stimuli. The task terminated when all three trials of the same sequence length were recalled incorrectly. Participants then proceeded to the backward recall, in which the instructions remained the same, except that the participants were required to reproduce the sequences in reverse order of presentation. The dependent variable was the longest sequence length accurately recalled in the forward and backward stages separately.

#### 2.2.4. Visuospatial Span

The computerized Visuospatial Span task, an adaptation of the Corsi Block-Tapping task [43], was employed to assess WM in the visuospatial modality. Nine purple squares were presented in a mixed array on the screen and sequentially changed color to yellow, one by one, for one second each in a predetermined order. In the forward recall, participants were instructed to replicate the sequence by clicking on the squares with a mouse in the same order. The task commenced with two square sequences, comprising two trials each. Upon successful recall in at least one trial, an additional square was incorporated into the subsequent sequence length. This procedure continued until the participant failed to reproduce two trials of the same length accurately. In the backward recall, the procedure remained the same, except participants were required to click on the squares in the reverse order of presentation. The dependent variable was the longest sequence length correctly recalled in the forward and backward stages separately.

#### 2.2.5. The Montreal Cognitive Assessment Task (MoCA)

The MoCA is a brief screening instrument designed to detect subtle cognitive impairments indicative of potential dementia in its early stages among adults. A score of 26 or above is suggestive of preserved cognitive functioning [44,45]. Its diagnostic utility has been validated across various linguistic and cultural populations, including Hebrew-speaking [46]. The MoCA was found to be more sensitive in detecting mild cognitive impairment than other tools [47,48]. Thus, it was judged to be an appropriate measure for the present study.

### 2.3. Procedure

Prospective participants who expressed interest underwent an initial screening conversation conducted by the examiner to assess their eligibility based on the predetermined criteria. Those meeting the inclusion requirements scheduled a meeting and were requested to complete the demographic questionnaire. Eligible participants then scheduled a meeting with the examiner, during which they provided informed consent and proceeded to complete the experimental tasks, which lasted approximately one hour.

### 2.4. Statistical Analysis

Initially, preliminary analyses were conducted, in which group differences in age and educational attainment were assessed through separate one-way analyses of variance (ANOVA), followed by Bonferroni post hoc tests. The distribution of gender across groups was compared using a chi-square test. To evaluate performance across span tasks and age groups, a 3 (Task) × 4 (Group) × 2 (Stage) mixed design repeated measures ANOVA was conducted, followed by Bonferroni post hoc tests where significant effects were found. Data analysis was carried out using SPSS version 29, with statistical significance determined at an alpha level of *p* < 0.05.

## 3. Results

Comparison of the four groups showed, as expected, a significant difference in the age variable (*F*(3,126) = 3031.96, *p* < 0.001, η_p_^2^ = 0.99). However, analysis of the groups showed a nonsignificant result for the years of education variable (*F*(3,126) = 2.52, *p* = 0.06; see Table 1 for the demographic variables of each group, and refer to Table 2 for descriptive data of the WM task scores by age group and stage).

**Figure 1:**
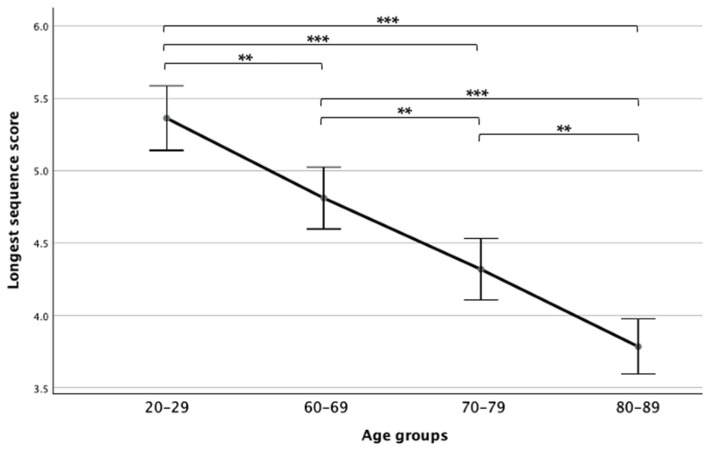
Means and standard deviations of the longest sequence scores in the four age groups. Note. ** *p* < 0.01; *** *p* < 0.001.

Comparing the four age groups showed a main effect (*F*(3,125) = 39.47, *p* < 0.001, η_p_^2^ = 0.48), while a Bonferroni post hoc test revealed that the 80–89 age group performed significantly worse than all other groups and the 20–29 age group performed significantly better than all age groups. Additionally, the 60–69 age group performed significantly better than the 70–79 age group (see Figure 1).

**Figure 2:**
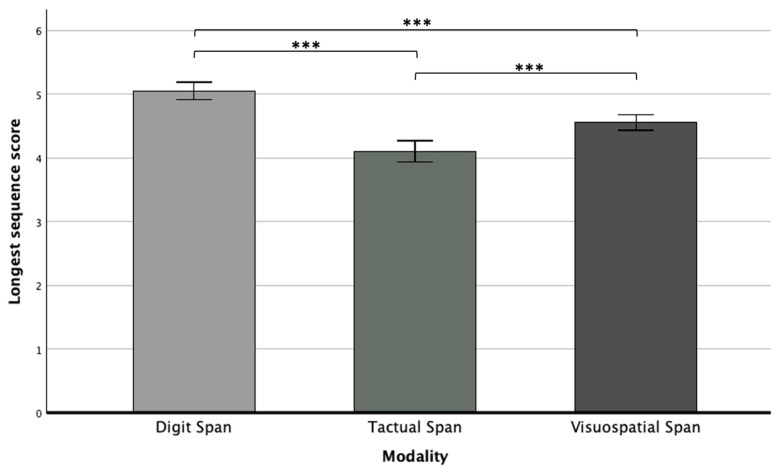
Means and standard deviations of the longest sequence scores in the Digit, Tactual, and Visuospatial Spans. Note. *** *p* < 0.001.

In addition, we found a main effect for modality (*F*(2,250) = 22.6, *p* < 0.001, η_p_^2^ = 0.15), whereas a Bonferroni post hoc test showed that the Digit Span was performed significantly better than the other two span tasks, and the Tactual Span was performed worse compared to the Digit and Visuospatial Spans (see Figure 2). Furthermore, a significant main effect for stage was revealed (*F*(1,125) = 55.48, *p* < 0.001, η_p_^2^ = 0.30), as performance in the forward stage (*M* = 4.93, *SD* = 0.06) was significantly better than in the backward stage (*M* = 4.21, *SD* = 0.06).

**Figure 3:**
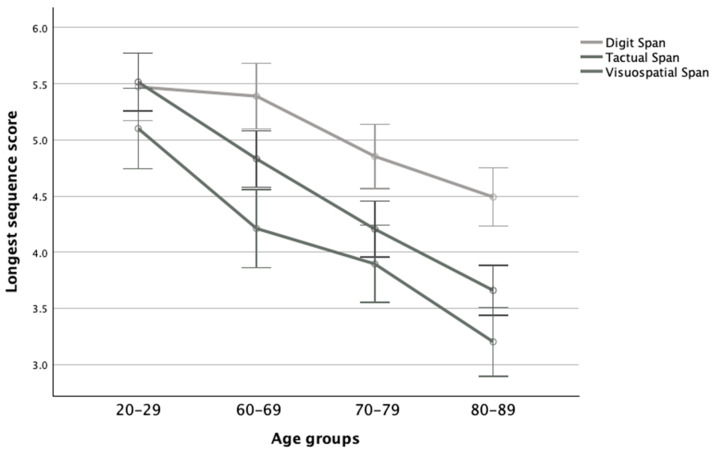
Means and standard deviations of the span tasks’ longest sequence scores in the four age groups.

There was also a significant interaction effect between the age groups and modality (*F*(6,250) = 3.33, *p* = 0.004, η_p_^2^ = 0.07), while further post hoc analyses revealed that for the 20–29 age group there were no significant differences among all modalities. Contrarily, the elderly groups performed significantly better on the Digit Span than the other modality tasks, while in the 60–69 and 80–89 age groups, the Tactual Span performed the worst. Moreover, in the verbal modality, the difference between the 20–29 and 60–69 age groups was nonsignificant, as was the difference between the 60–69 and 70–79 age groups, while the 80–89 age group performed significantly worse than other groups. However, in the tactile and visuospatial modalities, the 20–29 group performed significantly better than the other groups, while the 80–89 group performed substantially worse than the other age groups (see Figure 3).

**Figure 4:**
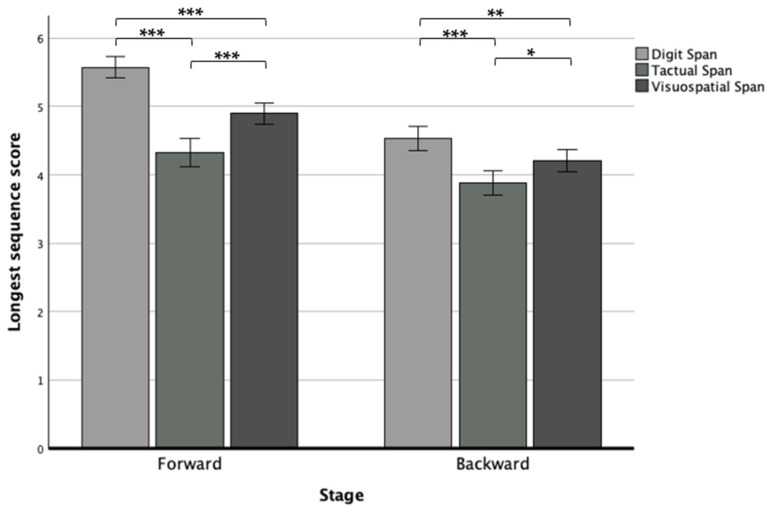
Means and standard deviations of the forward and backward longest sequence scores in the Digit, Tactual, and Visuospatial Spans. Note. * *p* < 0.05; ** *p* < 0.01; *** *p* < 0.001.

We found another interaction effect between modality and stage (*F*(2,250) = 7.41, *p* < 0.001, η_p_^2^ = 0.06), in which the post hoc revealed that in both stages, the Digit Span performed significantly better than the other modalities, and the Tactual Span performed worse compared to the other span tasks by showing in the forward stage, significantly larger differences than in the backward stage (see Figure 4).

In contrast, the interaction between the age groups and stage was not significant (*F*(3,125) = 1.68, *p* = 0.17), nor was the interaction between modality, age group, and stage (*F*(6,250) = 0.77, *p* = 0.59).

## 4. Discussion

The main purpose of this research was to explore the differential effect of aging on WM performance across the verbal, visuospatial, and tactile modalities in the storage and manipulation components.

We found a main effect for modality, with Digit Span task performance being better than that of the other modalities and Tactual Span task performance being the worst, thus indicating that verbal is the most skilled WM modality of the three while tactile is the least. This aligns with studies revealing that the frequency of daily usage may elucidate the observed modality-specific performance trends in WM [14,33,49], similar to previous studies employing tactile WM with broader investigations [50,51].

In further support of such a view, Bliss & Hämäläinen [52] have suggested that performance discrepancies between tactile and visual modalities may be due to participants’ inexperience with tactile information in WM tasks. Several recent studies have shown that WM performance in sensory-deprived individuals is influenced more by experience in the modality than by merely the intact sense [33,53,54].

An alternative explanation for the tactile WM performance deficit may involve the complexities of finger localization, which can delay the storage and manipulation of tactile information. Higher-order cognitive processes required for accurate touch localization contribute to this phenomenon. Tamè et al. [55] showed that mislocalization of tactile stimuli on fingers is influenced by both finger identity and spatial proximity, indicating that multiple factors impact performance on tactile tasks, which highlights the complexity of tactile information processing and its effects on WM function [40].

Another finding of the current study was a main effect for age, showing that the youngest group (20–29 years) outperformed all older groups, with the oldest group (80–89 years) performing the worst. These results align with studies demonstrating a general decline in WM capacity with age [8,10,12,17,18,56], which is attributed to alterations in neural structures, particularly the gray and white matter in the frontal cortex [57,58]. Costello and Buss [59] have shown that older adults exhibit more diffuse and attenuated neural activity due to reduced intracortical inhibition, resulting in broader and weaker neural signals. They were supported by imaging studies showing under-activity in primary visual processing areas in older adults compared to younger individuals [24].

The interaction effect between age groups and modality underscores the complexity of WM decline. For the 20–29 age group, there were no significant differences between modalities, but in the elderly groups, the Digit Span was performed significantly better than the other modality tasks, while Tactual Span performance was the worst in the 60–69 and 80–89 age groups. Enhanced performance in the verbal domain suggests that verbal information is more resilient than visuospatial information with age, while tactile WM is the most sensitive to it.

The above finding supports aging studies indicating a divergence between verbal, visuospatial, and tactile modalities [10,11,34], explained by either greater deterioration in the right hemisphere compared to the left (i.e., right hemi-aging model; [21]) or differentiation in daily use [49,60].

The interaction between modality and stage highlights the differential impact of the type of modality on WM storage and manipulation components. In all three modalities, the forward stage was performed better than the backward stage, thus corroborating Heled’s [40] findings of WM manipulation being more demanding in the three modalities. Consequently, this supports the claim that forward recall represents a passive retrieval mechanism, while backward recall is more demanding and more active in nature [61,62].

Regarding modality, in both stages, the Digit Span was performed significantly better than the tasks for the other modalities, while the Tactual Span was performed the worst, showing greater differences in the forward stage. The limited storage capacity of tactile WM compared to the other modalities may explain the observed phenomenon. Interestingly, this gap narrows upon manipulation, as the other modalities show a steeper performance decline compared to tactile WM, which decreases less, potentially due to its initially lower capacity. This pattern is in line with the observations of others who found no significant differences between tactile and verbal WM in the backward stage [33,34].

The current study has several limitations. The cross-sectional design limits causal inferences about WM decline over time, making longitudinal studies more suitable for evaluating modality-specific WM deterioration in older adults. Additionally, the reliance on span tasks, though common, may not sensitively capture manipulation performance. Incorporating a broader range of WM assessment tools, including complex span tasks, could provide a more comprehensive understanding of WM functioning in older adults. Finally, we did not collect specific data on participants’ cultural backgrounds. Thus, further studies should address this issue by considering the influence of cultural factors on WM performance across different modalities.

In conclusion, our study reveals nuanced age-related changes across modalities and process levels, providing a more accurate representation of WM performance. More specifically, the findings demonstrate that while verbal WM performance remains relatively stable with age, tactile and visuospatial modalities exhibit significant declines, with tactile WM showing the weakest performance. Consequently, this may indicate that there is a modality-specific nature of WM structure, suggesting that each WM modality may possibly represent a distinct system. Therefore, our study emphasizes the importance of modality-specific assessments for a comprehensive understanding of WM capacity in older adults. Interventions targeting cognitive decline could focus on preserving verbal WM while improving visuospatial and tactile WM, particularly in manipulation functioning.

## Figures and Tables

**Table 1:** Means and standard deviations (in parentheses) of participants’ ages and years of education, divided into age groups.

	20–29	60–69	70–79	80–89
Age	24.6 (2.88)	64.2 (2.77)	75.23 (2.12)	83.82 (2.86)
Years of education	13.93 (1.28)	13.83 (1.98)	14.71 (2.03)	13.51 (1.87)

**Table 2:** Means and standard deviations (in parentheses) of participants’ WM task scores by age groups and stage.

	20–29	60–69	70–79	80–89
Digit Span fw	6.13 (0.93)	5.87 (1.01)	5.26 (0.85)	5.05 (0.85)
Digit Span bw	4.87 (1.20)	4.93 (0.87)	4.42 (0.95)	3.90 (0.91)
Tactual Span fw	5.30 (1.26)	4.57 (0.81)	3.94 (1.43)	3.49 (1.07)
Tactual Span bw	4.93 (1.01)	3.87 (1.01)	3.84 (1.24)	2.90 (0.85)
Visuospatial Span fw	5.97 (0.81)	5.27 (0.24)	4.45 (1.09)	3.92 (0.84)
Visuospatial Span bw	5.17 (1.08)	4.43 (0.77)	3.90 (0.70)	3.33 (0.98)

Note. fw = forward; bw = backward.

## Data Availability

All data, analysis code, and research materials are available upon reasonable request from the corresponding author.

## Abbreviations

Working memory—WM.

## References

[1] Baddeley A.D. (2021). Developing the concept of working memory: The role of neuropsychology. Arch. Clin. Neuropsychol..

[2] Courtney S.M. (2022). Working memory is a distributed dynamic process. Cogn. Neurosci..

[3] Van Ede F., Nobre A.C. (2023). Turning attention inside out: How working memory serves behavior. Annu. Rev. Psychol..

[4] Diamond A. (2014). Understanding executive functions: What helps or hinders them and how executive functions and language development mutually support one another. Perspect. Lang. Lit..

[5] Vernucci S., Aydmune Y., Andrés M.L., Burin D.I., Canet-Juric L. (2021). Working memory and fluid intelligence predict reading comprehension in school-age children: A one-year longitudinal study. Appl. Cogn. Psychol..

[6] Belacchi C., Artuso C., Palladino P. (2022). Semantic long-term memory and verbal working memory performance: How does their relationship change with age?. Cogn. Dev..

[7] Forsberg A., Blume C.L., Cowan N. (2021). The development of metacognitive accuracy in working memory across childhood. Dev. Psychol..

[8] Krogsrud S.K., Mowinckel A.M., Sederevicius D., Vidal-Piñeiro D., Amlien I.K., Wang Y., Sørensen Ø., Walhovd K.B., Fjell A.M. (2021). Relationships between apparent cortical thickness and working memory across the lifespan-effects of genetics and socioeconomic status. Dev. Cogn. Neurosci..

[9] Lugtmeijer S., de Haan E.H., Kessels R.P. (2019). A comparison of visual working memory and episodic memory performance in younger and older adults. Aging Neuropsychol. Cogn..

[10] Swanson H.L. (2017). Verbal and visual-spatial working memory: What develops over a life span?. Dev. Psychol..

[11] Alloway T.P., Alloway R.G. (2013). Working memory across the lifespan: A cross-sectional approach. J. Cogn. Psychol..

[12] Borella E., Carretti B., De Beni R. (2008). Working memory and inhibition across the adult life-span. Acta Psychol..

[13] Cansino S., Hernández-Ramos E., Estrada-Manilla C., Torres-Trejo F., Martínez-Galindo J.G., Ayala-Hernández M., Gómez-Fernández T., Osorio D., Cedillo-Tinoco M., Garcés-Flores L. (2013). The decline of verbal and visuospatial working memory across the adult life span. Age.

[14] D’Antuono G., Maini M., Marin D., Boccia M., Piccardi L. (2022). Effect of ageing on verbal and visuo-spatial working memory: Evidence from 880 individuals. Appl. Neuropsychol. Adult.

[15] Vecchi T., Richardson J., Cavallini E. (2005). Passive storage versus active processing in working memory: Evidence from age-related variations in performance. Eur. J. Cogn. Psychol..

[16] Bisiacchi P.S., Borella E., Bergamaschi S., Carretti B., Mondini S. (2008). Interplay between memory and executive functions in normal and pathological aging. J. Clin. Exp. Neuropsychol..

[17] Kumar N., Priyadarshi B. (2013). Differential effect of aging on verbal and visuo-spatial working memory. Aging Dis..

[18] Park D.C., Lautenschlager G., Hedden T., Davidson N.S., Smith A.D., Smith P.K. (2002). Models of visuospatial and verbal memory across the adult life span. Psychol. Aging.

[19] Fournet N., Roulin J.-L., Vallet F., Beaudoin M., Agrigoroaei S., Paignon A., Dantzer C., Desrichard O. (2012). Evaluating short-term and working memory in older adults: French normative data. Aging Ment. Health.

[20] Goldstein G., Shelly C. (1981). Does the right hemisphere age more rapidly than the left?. J. Clin. Exp. Neuropsychol..

[21] Jockwitz C., Caspers S., Lux S., Jütten K., Schleicher A., Eickhoff S.B., Amunts K., Zilles K. (2017). Age-and function-related regional changes in cortical folding of the default mode network in older adults. Brain Struct. Funct..

[22] Payer D., Marshuetz C., Sutton B., Hebrank A., Welsh R.C., Park D.C. (2006). Decreased neural specialization in old adults on a working memory task. Neuroreport.

[23] Reuter-Lorenz P.A. (2012). Cognitive neuropsychology of the aging brain. Cognitive Aging.

[24] Kirova A.M., Bays R.B., Lagalwar S. (2015). Working memory and executive function decline across normal aging, mild cognitive impairment, and Alzheimer’s disease. BioMed Res. Int..

[25] Giannouli V., Kolev V., Yordanova J. (2019). Is there a specific Vivaldi effect on verbal memory functions? Evidence from listening to music in younger and older adults. Psychol. Music.

[26] Giannouli V., Yordanova J., Kolev V. (2024). Can Brief Listening to Mozart’s Music Improve Visual Working Memory? An Update on the Role of Cognitive and Emotional Factors. J. Intell..

[27] Mellet E., Tzourio N., Crivello F., Joliot M., Denis M., Mazoyer B. (1996). Functional anatomy of spatial mental imagery generated from verbal instructions. J. Neurosci..

[28] Wingfield A., Stine E.A., Lahar C.J., Aberdeen J.S. (1988). Does the capacity of working memory change with age?. Exp. Aging Res..

[29] Danovitch J.H. (2019). Growing up with Google: How children’s understanding and use of internet-based devices relates to cognitive development. Hum. Behav. Emerg. Technol..

[30] Fougnie D., Zughni S., Godwin D., Marois R. (2015). Working memory storage is intrinsically domain specific. J. Exp. Psychol. Gen..

[31] Gallace A., Spence C. (2014). In Touch with the Future: The Sense of Touch from Cognitive Neuroscience to Virtual Reality.

[32] Heled E., Rotberg S., Yavich R., Hoofien A.D. (2021). Introducing the tactual span: A new task for assessing working memory in the teactile modality. Assessment.

[33] Heled E., Israeli R., Margalit D. (2022). Working memory development in different modalities in children and young adults. J. Exp. Child Psychol..

[34] Heled E., Levi O. (2024). Aging’s Effect on Working Memory—Modality Comparison. Biomedicines.

[35] van Dam W.O., Decker S.L., Durbin J.S., Vendemia J.M., Desai R.H. (2015). Resting state signatures of domain and demand-specific working memory performance. Neuroimage.

[36] Vergauwe E., Camos V., Barrouillet P. (2014). The impact of storage on processing: How is information maintained in working memory?. J. Exp. Psychol. Learn. Mem. Cogn..

[37] Saults J.S., Cowan N. (2007). A central capacity limit to the simultaneous storage of visual and auditory arrays in working memory. J. Exp. Psychol. Gen..

[38] Nicholas J. (2010). From Active Touch to Tactile Communication: What’s Tactile Cognition Got to do with It?.

[39] Gignac G.E., Kovacs K., Reynolds M.R. (2018). Backward and forward serial recall across modalities: An individual differences perspective. Personal. Individ. Differ..

[40] Heled E. (2024). Forward versus backward recall: Modality testing. Appl. Neuropsychol. Adult.

[41] Faul F., Erdfelder E., Buchner A., Lang A.-G. (2009). Statistical power analyses using G* Power 3.1: Tests for correlation and regression analyses. Behav. Res. Methods.

[42] Wechsler D. (1997). Wechsler Memory Scale.

[43] Corsi P.M. (1972). Human Memory and the Medial Temporal Region of the Brain. Ph.D. Thesis.

[44] Nasreddine Z.S., Phillips N.A., Bédirian V., Charbonneau S., Whitehead V., Collin I., Cummings J.L., Chertkow H. (2005). The Montreal Cognitive Assessment, MoCA: A brief screening tool for mild cognitive impairment. J. Am. Geriatr. Soc..

[45] Yang T., Su X., Allen R.J., Ye Z., Jia L. (2022). Improving older adults’ ability to follow instructions: Benefits of actions at encoding and retrieval in working memory. Memory.

[46] Lifshitz M., Dwolatzky T., Press Y. (2012). Validation of the Hebrew version of the MoCA test as a screening instrument for the early detection of mild cognitive impairment in elderly individuals. J. Geriatr. Psychiatry Neurol..

[47] Aiello E.N., Pasotti F., Appollonio I., Bolognini N. (2022). Trajectories of MMSE and MoCA scores across the healthy adult lifespan in the Italian population. Aging Clin. Exp. Res..

[48] Ciesielska N., Sokołowski R., Mazur E., Podhorecka M., Polak-Szabela A., Kędziora-Kornatowska K. (2016). Is the Montreal Cognitive Assessment (MoCA) test better suited than the Mini-Mental State Examination (MMSE) in mild cognitive impairment (MCI) detection among people aged over 60? Meta-analysis. Psychiatr. Pol..

[49] Heled E., Ohayon M., Oshri O. (2022). Working memory in intact modalities among individuals with sensory deprivation. Heliyon.

[50] Cattaneo Z., Vecchi T. (2008). Supramodality effects in visual and haptic spatial processes. J. Exp. Psychol. Learn. Mem. Cogn..

[51] Katus T., Eimer M. (2018). Independent attention mechanisms control the activation of tactile and visual working memory representations. J. Cogn. Neurosci..

[52] Bliss I., Hämäläinen H. (2005). Different working memory capacity in normal young adults for visual and tactile letter recognition task. Scand. J. Psychol..

[53] Heled E., Ohayon M. (2021). Visuospatial and tactile working memory in individuals with congenital deafness. J. Deaf Stud. Deaf Educ..

[54] Heled E., Oshri O. (2023). Validation of the Tactual Span in individuals with congenital and acquired blindness. Br. J. Vis. Impair..

[55] Tamè L., Wühle A., Petri C.D., Pavani F., Braun C. (2017). Concurrent use of somatotopic and external reference frames in a tactile mislocalization task. Brain Cogn..

[56] Fandakova Y., Sander M.C., Werkle-Bergner M., Shing Y.L. (2014). Age differences in short-term memory binding are related to working memory performance across the lifespan. Psychol. Aging.

[57] Kennedy K.M., Raz N. (2009). Aging white matter and cognition: Differential effects of regional variations in diffusion properties on memory, executive functions, and speed. Neuropsychologia.

[58] Montembeault M., Joubert S., Doyon J., Carrier J., Gagnon J.-F., Monchi O., Lungu O., Belleville S., Brambati S.M. (2012). The impact of aging on gray matter structural covariance networks. Neuroimage.

[59] Costello M.C., Buss A.T. (2018). Age-related decline of visual working memory: Behavioral results simulated with a dynamic neural field model. J. Cogn. Neurosci..

[60] Mahrer P., Miles C. (2002). Recognition memory for tactile sequences. Memory.

[61] Heled E. (2024). Laterality in tactile working memory: The one-hand version of the Tactual Span. J. Neuropsychol..

[62] Norris D., Hall J., Gathercole S.E. (2019). How do we perform backward serial recall?. Mem. Cogn..

